# Nonlinear Energy Evolution Characteristics of Diorite Examined by Triaxial Loading–Unloading and Acoustic Emission Tests

**DOI:** 10.3390/ma15186434

**Published:** 2022-09-16

**Authors:** Xuexu An, Zhiping Hu, Liang Zhang, Anlong Liu, Yonghui Zhang, Fangtao Li

**Affiliations:** 1 School of Civil Engineering, Chang’an University, Xi’an 710061, China; 2 School of Civil Engineering, Xi’an University of Science and Technolog, Xi’an 710054, China; 3 Machinery Industry Survey, Design and Research Institute Co., Ltd., Xi’an 710005, China

**Keywords:** damage evolution, loading and unloading, acoustic emission, period-doubling bifurcation, chaos

## Abstract

Acoustic emission (AE) is often accompanied by the propagation of internal microcracks in loaded rock samples, and it essentially reflects microinstability phenomena driven by energy redistribution under stress. In this paper, loading and unloading tests were carried out to investigate the internal nonlinear damage evolution characteristics of diorite samples under different unloading confining-pressure rates. The nonlinear mechanical characteristics of the strain energy sequence of diorite were studied by applying nonlinear dynamics and basic chaos theory and MATLAB software. Moreover, the evolution characteristics of AE counts and AE energy of rock samples were investigated, and their microcrack-propagation modes were analyzed based on the *RA*–*AF* scatter distribution of AE and a two-dimensional Gaussian mixture model. Finally, according to the evolution characteristics of energy and AE, the nonlinear damage evolution mechanism of diorite under loading and unloading conditions was revealed. The results show that, before the loading and unloading peak strength, when the strain-energy-promotion coefficient, *r*, is equal to 1 or changes in the ranges of 1–3, 3–3.57, and ≥3.57, the strain-energy evolution of diorite presents the characteristics of supercritical stability, nonlinear stability, period-doubling stability, and chaos, respectively. Meanwhile, the greater the rate of the unloading confining pressure, the earlier the period-doubling bifurcation and chaotic mechanical behavior will occur. After loading and unloading peak strength, the sudden decrease of high-density AE counts and AE energy or the sudden transition of the strain-energy-promotion coefficient from >0 to <0 can be used as an important criterion for the complete failure of rock samples.

## 1. Introduction

Rocks are extremely heterogeneous materials, such that a large number of microcracks form within them under long-term in situ stress. During the loading process, stress concentrations and strain energy accumulation initially develop around microcracks in rocks. When the stress concentration exceeds its strength, these microcracks begin to develop further in order to reduce the stress concentration. Simultaneously, a part of the stored strain energy is released in the form of elastic waves; this induces the phenomenon of acoustic emission (AE) [1,2]. The AE of a loaded rock is an intuitive reflection of the evolution of its internal microcracks, reflecting microinstability phenomena driven by energy redistribution. Therefore, studying the evolution characteristics of rock energy and AE under complex stress paths is helpful to improve our understanding of the mechanisms governing rock-damage evolution.

The damage process of loaded rock is closely related to its internal energy flow and transformation [3]. Xie et al. [4] analyzed the relationship between energy dissipation, damage, energy release, and failure throughout the processes of rock deformation and failure, proposing a rock-strength-failure criterion based on energy dissipation and release theory. Li [5] investigated the relationship between material failure and internal energy, finding that energy dissipation and strain energy redistribution associated with a new surface reduces material strength. Liu et al. [6] studied the influence of water saturation on the energy evolution of mudstone via uniaxial compression, showing that water saturation weakens energy absorption and release of rock and enhances the energy dissipation of rock. Wang et al. [7] explored the energy-damage evolution mechanism of jointed rock masses from the perspective of energy through uniaxial compression tests and established the strength-failure criterion of non-cross-jointed rock masses. It was concluded that the energy storage in rock was significantly weakened by its internal non-cross joints. The damage and failure of rocks under loading and unloading conditions have also received considerable attention [8,9], and some true triaxial and conventional triaxial tests under complex loading and unloading stress-path conditions have been used to explore the damage and failure mechanisms of rocks [10], and the evolution of different energy indicators have been discussed [11]. These results have significantly improved our understanding of energy evolution mechanisms in the damage and failure processes of loaded rock.

AE is a non-destructive testing technique and has been widely used in the field of rock mechanics to become one of the most effective methods for revealing internal fracture behavior, failure mode, and damage evolution of loaded rock [12,13,14,15]. For example, Eberhardt et al. [16] studied the AE characteristics during the failure of granite by using AE detection technology, finding that the onset of AE activity closely corresponds to the initiation of stress-induced cracks in the rock. Lokajíček et al. [17] performed AE tests on granite and granulite, revealing that their crack-initiation stresses are 45% and 85% of their peak strength, respectively. Wang et al. [18] conducted AE tests on red sandstone, showing that the AE energy corresponding to shear failure was significantly greater than that corresponding to tensile failure. Zhang et al. [19] investigated the damage evolution and failure mode of coals under loading and unloading conditions by using the space–time dimension cluster analysis method and AE positioning technology. The results show that the AE events of short, middle, and long link reflected the local damage, overall, and random damage processes of coals, respectively. Additionally, AE events, AE energy, AE counts, and other AE parameters are used to characterize the internal damage evolution of rocks [20,21,22,23,24]. Thomas et al. [25] studied AE evolution characteristics during the damage evolution of granite via loading and unloading triaxial tests. The results demonstrated that, with the increase of freeze–thaw cycle times, the acoustic emission events of rocks were more active and presented local high-density release characteristics. Song et al. [26] used AE technology to study the mesoscale damage characteristics of freeze–thaw limestone under uniaxial compression, revealing its progressive damage mechanisms. Yang et al. [27] applied AE and X-ray CT observations to study the influence of joint dip on the fracture evolution mechanisms of discontinuous jointed rock masses, revealing that tensile wing cracks were dominated for smaller joint angles and shear wing cracks were dominated for large joint angles during. Zhang et al. [28] characterized the damage processes affecting granite by using different AE characteristics measured during uniaxial compression, finding that the initial damage time inferred from AE events was earlier than the macroscopic deformation.

The abovementioned works from the literature have improved our understanding of the damage evolution mechanisms of rock; however, these existing research results mostly focus on the description of the evolution process of rock energy and AE parameters under different stress paths. For a heterogeneous rock with initial defects, the damage evolution is a complex nonlinear change from order to disorder, from equilibrium to non-equilibrium, and from certain to uncertain. In this study, the nonlinear mechanical behavior of the rock strain energy sequence data of diorite under different loading and unloading conditions was investigated by using basic nonlinear dynamics theory and chaos theory. Furthermore, the evolution characteristics of AE counts, AE energy, and the microfracture propagation mode are analyzed. Finally, the damage evolution mechanism of diorite is revealed according to the evolution characteristics of energy and AE.

## 2. Test Process

### 2.1. Rock-Specimen Preparation

**Figure 1:**
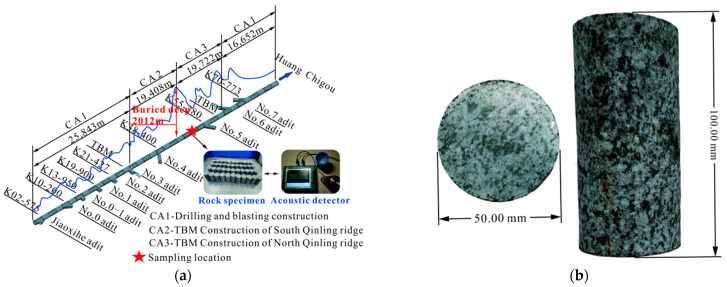
Sample position and size: (**a**) Position of rock sample source, (**b**) Rock sample size.

The diorite that was used in this test came from a deeply buried hard rock tunnel in the north of the Qinling Mountains, Shaanxi Province, China (Figure 1a), and is mainly composed of plagioclase, amphibole, and some minor dark minerals. The crystals in the rock exhibit a high degree of crystallization, and their shapes show various blocky structures of variable size. In the laboratory, a large number of cylinder-shaped samples, as shown in Figure 1b, were formed by drilling, cutting, and grinding. In order to reduce the influence of machine error and large microdefects on the test results, all samples were ground to ensure that the non-parallelism and diameter error of the upper and lower end faces was less than 0.02 mm. Subsequently, all rock samples were tested by using an RSM-SY5 (T) non-metallic acoustic detector (Wuhan Zhongyan Technology Co., Ltd., Wuhan, China) to remove rock samples with large acoustic dispersion phenomena.

### 2.2. Test Apparatus and Scheme

**Figure 2:**
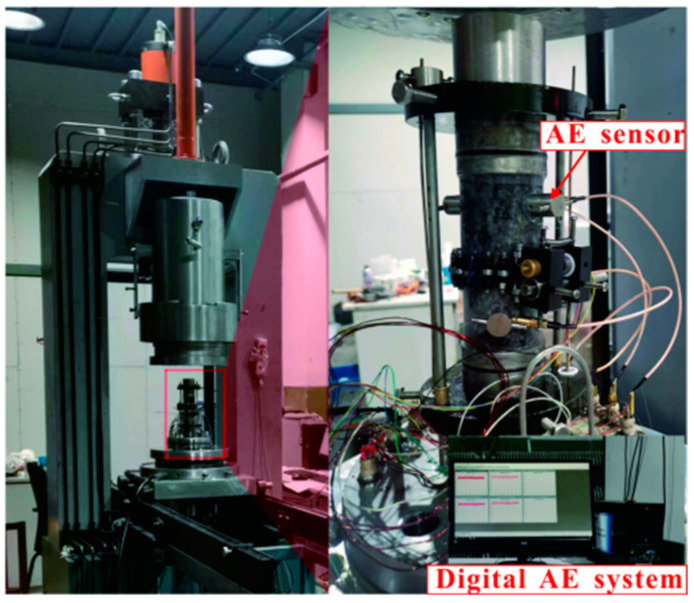
Loading and unloading test system.

The test apparatus is a SAS-2000 microcomputer-controlled rock triaxial test system (Figure 2) (Changchun Xinte testing machine Co., Ltd., Changchun, China). This apparatus is mainly composed of a pressurization system, a servo system, a control system, and a data-acquisition system; its maximum axial test load is 1500 KN, and the maximum confining pressure is 100 MPa. During the test, the system records the load, stress, displacement, and strain values of the studied rock sample in real time, simultaneously producing the load–displacement and stress–strain curves. The AE testing apparatus is a micro II express digital AE system with 32 channels, and the amplifier and threshold were set to 42 and 60 dB, respectively. The AE signals during the propagation of internal microcracks in loaded rock samples is acquired through four sensors fixed on the side surface of the rock sample and then transmitted to the AE control system after passing through the signal amplifier. Finally, these AE signals are recorded and displayed after being processed by the AE control system.

**Figure 3:**
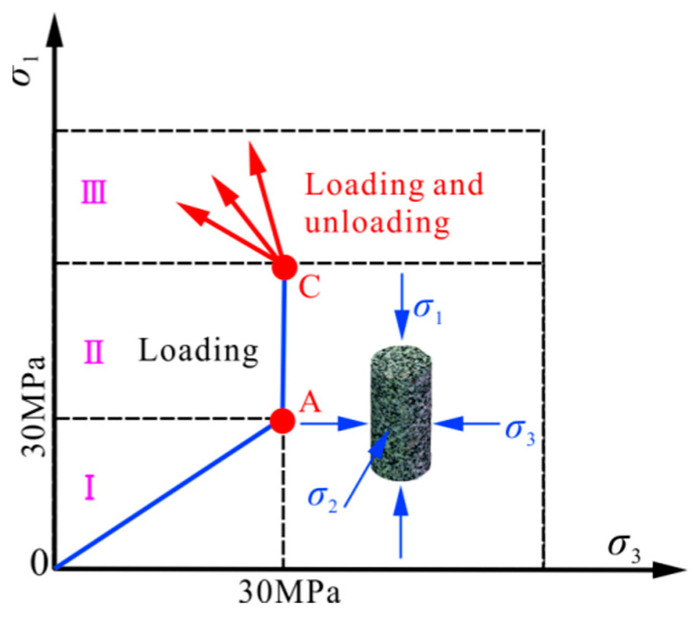
Schematic diagram of the loading and unloading pathways. Note: I, II and III represent the first, second and third stages of loading and unloading test, respectively. A and C are initial confining pressure point and initial point of unloading confining pressure, respectively.

The confining pressure of the loading and unloading test is 30 MPa; the peak strength of rock sample under the same confining pressure is 254.727 MPa; and its unloading confining-pressure rates are 0.008 MPa·s^−1^, 0.016 MPa·s^−1^, and 0.032 MPa·s^−1^, respectively. The test scheme is shown in Figure 3. The detailed scheme is as follows: (I) Increase the confining pressure and axial pressure at a rate of 0.05 MPa·s^−1^ to 30 MPa at point A. (II) Maintain the confining pressure, and then increase the axial pressure at a rate of 0.1 mm·min^−1^ through displacement control to 70% of the peak strength of the rock sample, i.e., point B. (III) Reduce the confining pressure at a predetermined unloading confining-pressure rate (0.008 MPa·s^−1^, 0.016 MPa·s^−1^, and 0.032 MPa·s^−1^), while maintaining a constant axial loading rate until failure of the rock sample.

## 3. Nonlinear Damage Characteristics of Diorite under Loading and Unloading Conditions

### 3.1. Energy Evolution Theory

During the loading and unloading process, the energy input from the test apparatus to the rock sample under hydrostatic pressure is expressed as *U*_0_; after increasing the axial stress difference, the energy input is expressed as *U*_1_, and the energy consumed generated by the circumferential deformation of the rock sample is expressed as *U*_3_. Then the total energy, *U*, in the rock sample can be expressed as shown below [29]:

(1) $$U=U_{1}+U_{3}+U_{0}$$

According to the theory of elasticity, the strain energy, *U*_0_ of a rock sample under hydrostatic pressure can be calculated by using Equation (2) [29]:

(2) $$U_{0}=\frac{3\left(1-2\mu \right)}{2E}{\left(\sigma_{3-o}\right)}^{2}$$

where *E* is the elastic modulus, which has a value corresponding to the slope of the straight-line section of the stress–strain curve; *μ* is the Poisson value; and *σ*_3-_*_o_* is the initial confining pressure.

The energy generated by the axial and radial stresses at any time, *t*, during the test can be obtained by using Equation (3):

(3) $$U_{1}=\int_{0}^{\varepsilon_{1t}}\sigma_{1}d\varepsilon \;U_{3}=2\int_{0}^{\varepsilon_{3t}}\sigma_{3}d\varepsilon_{3}$$

where *ε*_1*t*_ and *ε*_3*t*_ are the axial and circumferential strains of the rock sample at time, *t*, respectively; and *σ*_1_ and *σ*_3_ are the maximum and minimum principal stresses, respectively.

**Figure 4:**
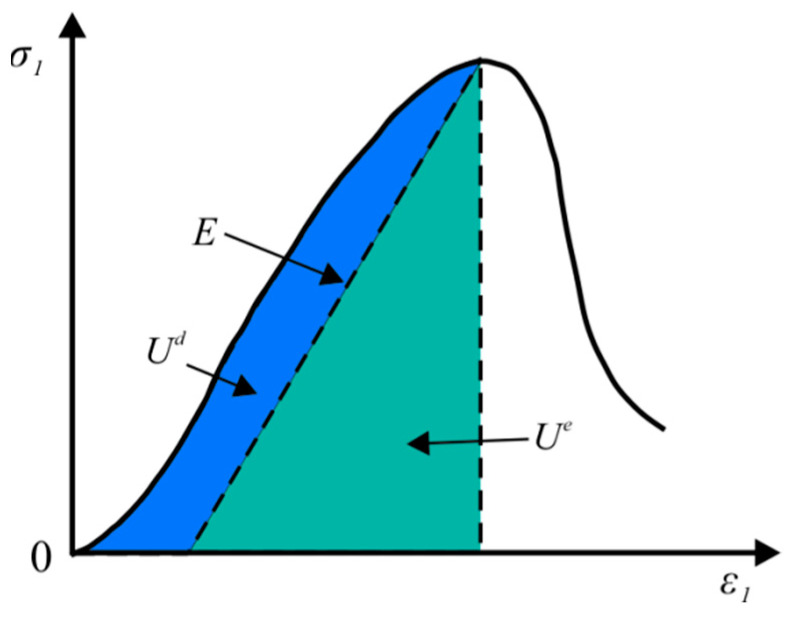
The elastic strain energy and dissipated energy in the rock.

If thermal energy is neglected in the energy exchange process of the rock sample, the total energy, *U*, given by the test apparatus to the rock sample can be considered to be mainly converted into elastic strain energy, *U^e^*, and dissipated energy, *U^d^* (Figure 4) [30]. Here, elastic strain energy is stored inside the rock in the form of releasable energy, while dissipative energy is consumed by internal damage during deformation. Equation (1) can thus be replaced by the following Equation (4):

(4) $$U=U^{e}+U^{d}$$

For the conventional triaxial loading and unloading test, the elastic strain energy can be calculated using Equation (5) [31]:

(5) $$U^{e}=\frac{1}{2E}\left[\sigma_{1}^{2}+2\sigma_{3}^{2}-2\mu \left(2\sigma_{1}\sigma_{3}+\sigma_{3}^{2}\right)\right]$$

For discrete experimental data, *U*_1_ and *U*_3_ generated by the axial stress, as well as the confining pressure, can be calculated by summing the areas of tiny rectangles. Then, the total energy *U* in Equation (1) can be expressed as follows using Equation (6) [31]:

(6) $$U=\overset{m}{\underset{i=1}{\sum }}\frac{1}{2}\left(\sigma_{1i}+\sigma_{1i-1}\right)△\varepsilon_{\left(1i\right)-\left(1i-1\right)}-\overset{m}{\underset{i=1}{\sum }}\left(\sigma_{3i}+\sigma_{3i-1}\right)△\varepsilon_{\left(3i\right)-\left(3i-1\right)}+\frac{3\left(1-2\mu \right)}{2E}{\left(\sigma_{3}^{0}\right)}^{2}$$

where *σ*_1*i*_, *σ*_1*i−*1_ and Δ*ε*_(__1*i*)−(1*i−*1)_ are the stress and strain difference of any adjacent two points in the axial stress–strain curve, respectively. Similarly, *σ*_3*i*_, *σ*_3*i*−1_ and Δ*ε*_(3*i*)−(3*i*−__1)_ are the stress and strain differences between any two adjacent points in the lateral stress–strain curve, respectively, and m is the total number of sample points.

According to Equations (4)–(6), the dissipation energy *U^d^* may be calculated using Equation (7) as follows:

(7) $$\begin{array}{l} U^{d}=\overset{m}{\underset{i=1}{\sum }}\frac{1}{2}\left(\sigma_{1i}+\sigma_{1i-1}\right)△\varepsilon_{\left(1i\right)-\left(1i-1\right)}-\overset{m}{\underset{i=1}{\sum }}\left(\sigma_{3i}+\sigma_{3i-1}\right)△\varepsilon_{\left(3i\right)-\left(3i-1\right)}+\frac{3\left(1-2\mu \right)}{2E}{\left(\sigma_{3}^{0}\right)}^{2}\\ -\frac{1}{2E}\left[\sigma_{1}^{2}+2\sigma_{3}^{2}-2\mu \left(2\sigma_{1}\sigma_{3}+\sigma_{3}^{2}\right)\right] \end{array}$$

### 3.2. Nonlinear Energy Evolution Characteristics

**Figure 5:**
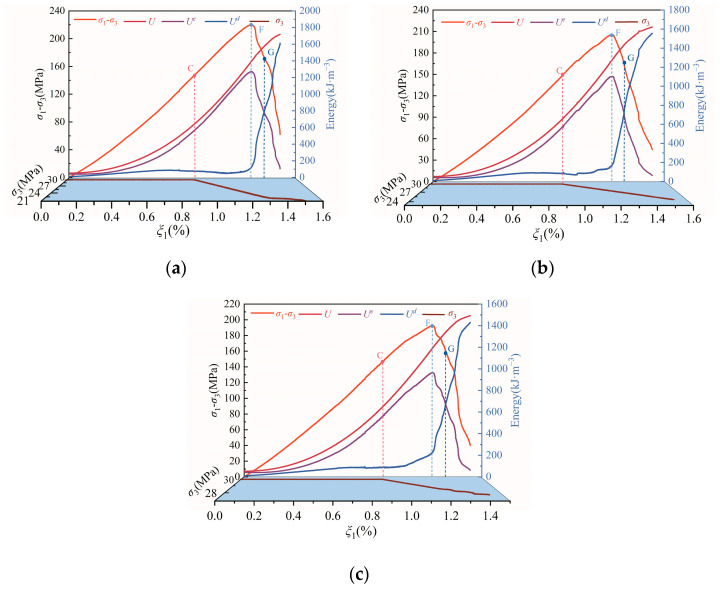
Energy evolution curves of diorite under different unloading confining-pressure rates: (**a**) 0.008 MPa·s^−1^, (**b**) 0.016 MPa·s^−1^, and (**c**) 0.032 MPa·s^−^^1^.

The propagation and evolution of microcracks in rock under loading and unloading is a progressive damage process driven by energy. Based on test data, the energy evolution curves of diorite under different unloading confining-pressure rates can be obtained by using Equations (5)–(7), as shown in Figure 5.

As shown in Figure 5, the curves of total and strain energy in the rock samples at the initial compression stage almost coincide, indicating that the hydrostatic pressure during the initial loading stage results in the closing of most of the initial microcracks in the rock sample, such that the energy dissipation at this stage is low. When the axial stress difference increases, the total energy and strain energy show an increasing upward concave trend under different unloading confining-pressure rates, whereas the dissipated energy first increases slowly and then gradually tends to become stable, implying that the internal damage to the rock sample is relatively small but that the strain energy rapidly accumulates during this process. After the initial stage of unloading confining pressure, i.e., after point C, the increment of the dissipated energy of the rock sample is small, but the increment of total energy and strain energy remains significant. This reflects the fact that the energy input from the axial direction of the rock sample is much greater than its dissipated energy. Following the continuous decrease of confining pressure and the increase of axial stress difference, the dissipated energy of the rock sample begins to increase gradually. When the axial stress difference exceeds the unloading peak stress, the dissipated energy begins to rise rapidly, while the elastic strain energy begins to decline rapidly. Before the G point of the stress–strain curve, although the bearing capacity of the rock sample decreases, the total energy still shows an increasing trend due to strong lateral constraints. After passing the G point, the total energy curve tends to gradually become stable, indicating the failure of the rock sample.

**Figure 6:**
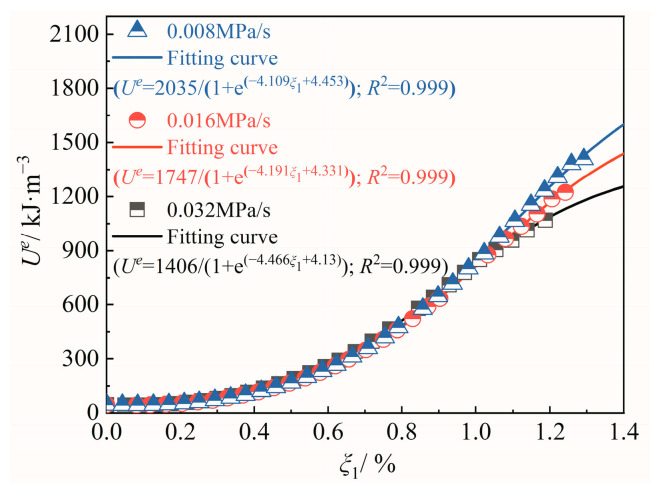
Relationship between pre-peak elastic strain energy of diorite and self-repression model under different unloading confining-pressure rates.

The above analysis shows that different energy evolution curves before the peak strength of diorite under different unloading confining-pressure rates exhibit nonlinear characteristics, whereas the strain-energy-evolution curves before the peak strength are best represented by the energy self-repression model in Equation (8); this model was proposed (as shown in Figure 6) by Wang et al. [32] as follows:

(8) $$U^{e}=\frac{\alpha }{1+e^{-r\varepsilon_{1}+c}}$$

where *α* reflects the ultimate strain energy storage capacity of a rock sample, considering that the self-repression of strain energy, *r*, reflects the promoting effect of strain energy without considering the self-repression of strain energy, and *c* is the integral constant.

By dividing both sides of Equation (8) by *α*, and then replacing *U^e^*/*α* with *η*, Equation (9) can be obtained as follows:

(9) $$\eta =\frac{U^{e}}{\alpha }=\frac{1}{1+e^{-r\varepsilon_{1}+c}}$$

Equation (10) can then be obtained by deriving Equation (9):

(10) $$\frac{d\eta }{d\varepsilon_{1}}=r\frac{1}{1+e^{-r\varepsilon_{1}+c}}\left(1-\frac{1}{1+e^{-r\varepsilon_{1}+c}}\right)=r\eta \left(1-\eta \right)$$

Equation (10) is a logistic mathematical model. Previous research [33] has shown that, when *r* increases from 1.0 to 3.0, the left value of the Equation (10) tends toward a stable value after multiple oscillations. When *r* increases from 3.0 to approximately 3.57, the left value of the Equation (10) produces a period-doubling bifurcation, i.e., from single period to multiperiod. When *r* is greater than 3.57, the left value of Equation (10) shows chaotic characteristics.

**Figure 7:**
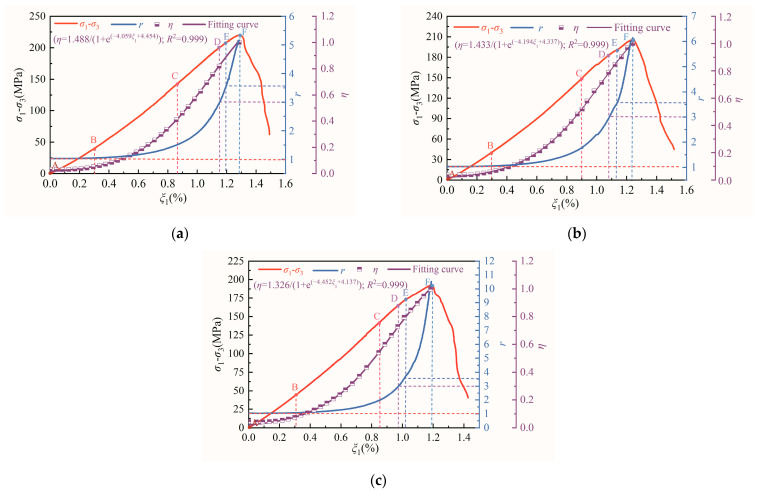
Chaotic mechanical phenomena of diorite strain energy accumulation under different unloading confining-pressure rates: (**a**) 0.008 MPa·s^−^^1^, (**b**) 0.016 MPa·s^−^^1^, and (**c**) 0.032 MPa·s^−^^1^.

In order to investigate the nonlinear damage characteristics of rock samples under different loading and unloading paths, the strain energy and axial strain data of diorite is brought into Equation (10) to obtain the nonlinear evolution curves of the pre-peak strain energy of diorite under different unloading confining-pressure rates, as shown in Figure 7.

Figure 7 shows that, for an axial strain ranging from 0.0% to 0.3%, increasing axial stress differences lead to an elastic strain-energy-promotion coefficient, *r*, that is almost unchanged, with a value approximately equal to 1. This indicates that the rock sample system is in a transcritical equilibrium state. The energy input by the test apparatus is rapidly transformed into internal elastic strain energy through internal microdeformation mechanisms, and almost no dissipative energy is formed during this process. When the axial-stress difference increases from point B to point D, *r* gradually increases from 1 to 3. During this process, the initial internal equilibrium state of the rock sample system is broken, and the energy input applied by the testing machine causes the rock-sample system to enter a state of repeated oscillation between equilibrium and non-equilibrium, which tends to become stable after several oscillations. This change can cause energy dissipation within the rock sample, but it mainly results in the accumulation of elastic strain energy. When the axial stress difference increases from point D to point E, *r* gradually increases from 3 to 3.57, implying that the rock sample system undergoes a period-doubling bifurcation oscillation state. This state causes continuous and predictable damage to the rock sample, thereby increasing its dissipated energy. When the axial-stress difference increases from point E to point F, *r* becomes greater than 3.57, and the rock-sample system enters a mixed state of order and chaos. This shows that the rock sample system is undergoing relatively complex energy conversion, during which damage to the sample begins to intensify.

**Figure 8:**
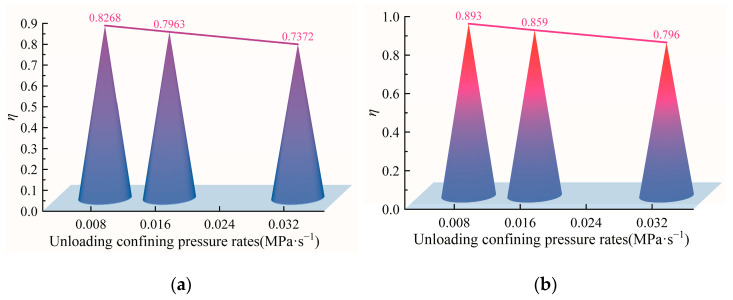
Effect of unloading confining-pressure rate on the nonlinear mechanical behavior of the diorite strain energy sequence: (**a**) critical values of period-doubling bifurcation; (**b**) chaotic critical values.

From the above analysis, it can be concluded that the evolution of strain energy before the peak strength of diorite under different unloading confining-pressure rates shows period-doubling bifurcation and chaotic mechanical behavior. To explore the influence of the unloading confining-pressure rate on the nonlinear mechanical behavior of rock samples, *η* (the ratio of strain energy to maximum strain energy) values corresponding to the thresholds of period-doubling bifurcation and chaotic phenomenon before the peak strength of diorite under different unloading confining-pressure rates in Figure 7 are shown in Figure 8.

It is clear from Figure 8 that increasing the unloading confining-pressure rates causes the *η* values corresponding to the thresholds of the period-doubling bifurcation and chaotic phenomenon before the peak strength of diorite to show a linear decreasing trend. This shows that the larger the unloading confining-pressure rate, the earlier the period-doubling bifurcation and chaotic mechanical behavior before the diorite peak occur. Such conditions are unfavorable to the stability of the rock system.

**Figure 9:**
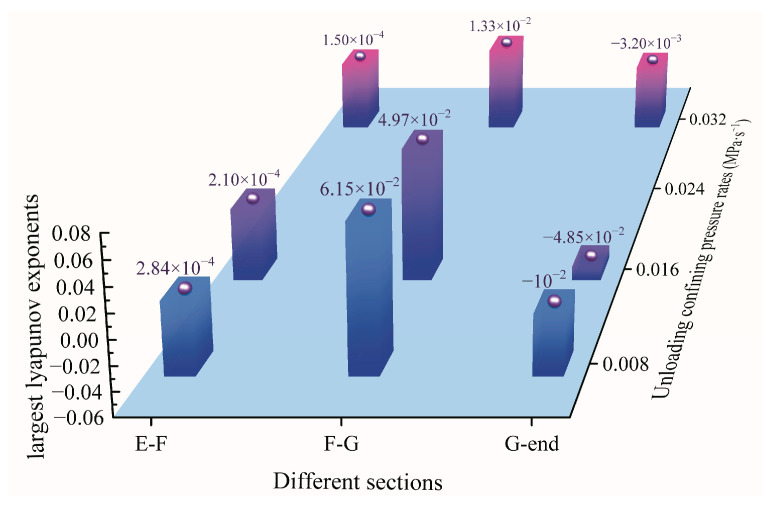
Maximum Lyapunov exponents in different damaged sections of diorite under variable unloading confining-pressure rates.

In order to further investigate the characteristics of the changing chaotic degree of the diorite strain energy sequence after entering the chaotic stage, the maximum Lyapunov exponents of the elastic strain energy sequence corresponding to the E–F, F–G, and G–end sections in the stress–strain curve were calculated. Before calculating the Lyapunov exponent, it is necessary to reconstruct a topological physical space equivalent to the original rock-sample system. In this paper, the more effective mutual information method and Gao method are used, such that the delay time interval and embedded dimension of the strain-energy sequence corresponding to the E–F, F–G, and G–end sections in the stress–strain curve are determined by the MATLAB program. The results of this operation are shown in Table 1. Based on these results, the maximum Lyapunov exponents of the different strain-energy sequence are obtained by the Wolf method, and the results are shown in Figure 9.

The maximum Lyapunov exponent is an important parameter for judging whether the nonlinear time sequence is a chaotic system and for evaluating its degree of chaos. Lyapunov exponents greater than 0 indicate a chaotic state, whereas Lyapunov exponents less than 0 denote a stable state. When the Lyapunov exponent is equal to 0, the material is considered to be in a critical state between stability and chaos. From Figure 9, it is found that, under different unloading confining-pressure rates, the E–F section before the peak strength of diorite is in a weak chaotic state, whereas the F–G section after the peak strength is in a strong chaotic state; the degree of chaos in this section decreases with increasing unloading confining-pressure rates. The G–end section is in an orderly steady state, indicating that the rock system in this section has completely failed.

### 3.3. AE Characteristics

**Figure 10:**
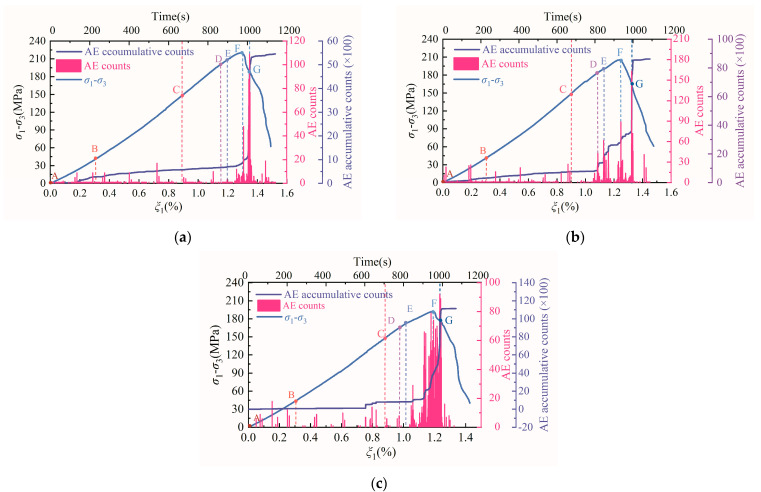
Evolution curves of AE counts and cumulative AE counts for diorite under different unloading confining-pressure rates: (**a**) 0.008 MPa·s^−1^, (**b**) 0.016 MPa·s^−1^, and (**c**) 0.032 MPa·s^−1^. Note: The meaning of A–G in the above figures is the same as that of A–G in Figure 5 and Figure 7.

**Figure 11:**
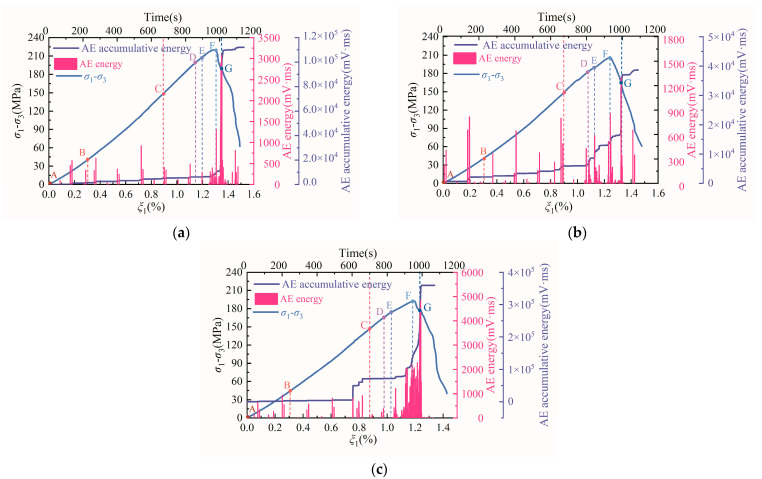
Evolution curves of AE energy and cumulative AE energy for diorite under different unloading confining-pressure rates: (**a**) 0.008 MPa·s^−1^, (**b**) 0.016 MPa·s^−1^, and (**c**) 0.032 MPa·s^−1^. Note: The meaning of A–G in the above figures is the same as that of A–G in Figure 5 and Figure 7.

Section 3.2 recounted the investigation of the nonlinear damage behavior of the strain-energy sequence of diorite. In the following, the AE characteristics of diorite under loading and unloading conditions are studied. Figure 10 shows the evolution curves of AE counts and cumulative AE counts for diorite under different unloading confining-pressure rates. Figure 11 shows the AE-energy and cumulative-AE-energy evolution curves of diorite under different unloading confining-pressure rates.

During loading and unloading, the closure, initiation, propagation, and penetration of microcracks throughout the rock sample system is accompanied by AE events. As shown in Figure 10 and Figure 11, before the unloading confining pressure (points A–C), the AE counts, cumulative AE counts, AE energy, and cumulative AE energy all changed slightly with the increasing axial-stress difference. At the initial stage of unloading confining pressure (points C–D) and during the period-doubling bifurcation D–E section, the AE counts, cumulative AE counts, AE energy, and cumulative AE energy also changed slightly, indicating that the internal damage of the rock-sample system is small in the A–E section of the rock sample. These characteristics show that the integrity of the rock in A–E section is good, proving its stable and orderly nonlinear dynamic behavior. As the axial-stress difference continues to increase, the AE counts, cumulative AE counts, AE energy, and cumulative AE energy begin to increase significantly in the E–F section, reflecting an increase in the internal damage of the rock sample. When the axial-stress difference crosses the unloading peak stress F point and enters the post-peak F–G section, the AE counts, cumulative AE counts, AE energy, and cumulative AE energy show the characteristics of rapid increase, implying intense internal damage. When the post-peak stress–strain curve of the rock sample falls to the G–end section, the AE counts, cumulative AE counts, AE energy, and cumulative AE energy begin to plummet, indicating that the rock sample system has completely failed. These AE evolution characteristics indicate that the rock-sample system exhibits a chaotic state in the E–G section and a steady state in the G–end section.

**Figure 12:**
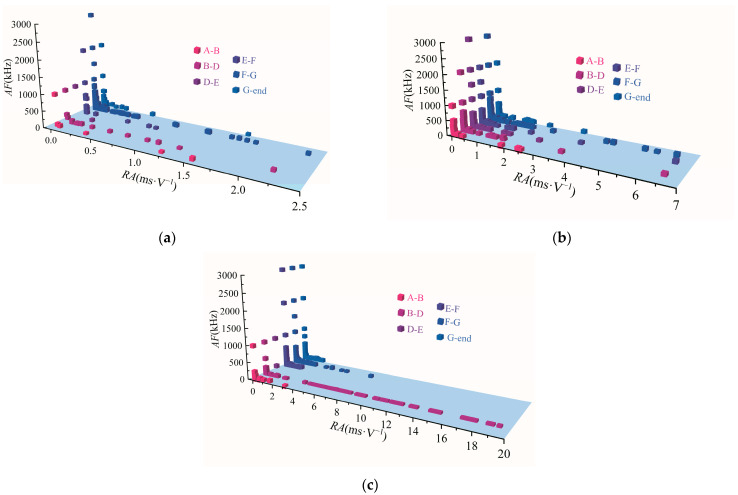
*RA*–*AF* distributions in different damaged sections of diorite under variable unloading confining-pressure rates: (**a**) 0.008 MPa·s^−1^, (**b**) 0.016 MPa·s^−1^, and (**c**) 0.032 MPa·s^−1^.

AE counts and AE energy can better show the damage-evolution characteristics of rocks, but they cannot indicate the evolution mode of internal microcracks. In the following section, *RA* and *AF* on different sections of diorite under different unloading confining-pressure rates are analyzed. *AF* denotes the ratio of AE counts to its duration, whereas *RA* indicates the ratio of AE rise time to its amplitude. Many previous studies have also found that AE has the characteristics of short rise time and duration, large amplitude, and high counts when tensile cracks are generated in rock samples; in other words, the *AF* value is large, and the *RA* value is small, while AE has the opposite characteristics when shear cracks are generated [34,35]. Using AE test data, the *RA*–*AF* scatter distributions of diorite under different unloading confining-pressure rates were determined, and they are shown in Figure 12.

As shown in Figure 12, under different unloading confining-pressure rates, the *AF*–*RA* scatter distributions of A–B, B–D, D–E, E–F, F–D, and G–end sections of diorite are mainly distributed at 0–1000 kHz and 0–2 ms·V^−1^, but during unloading, the E–F, F–D, G–end sections exhibit some larger *AF* parameters. It can be seen that the internal crack propagation of diorite in the process of loading and unloading under high confining-pressure condition is mainly dominated by tension–shear mixing. Moreover, during the unloading process, some tensile cracks with high *AF* parameters are induced inside the rock due to the unloading effect.

**Figure 13:**
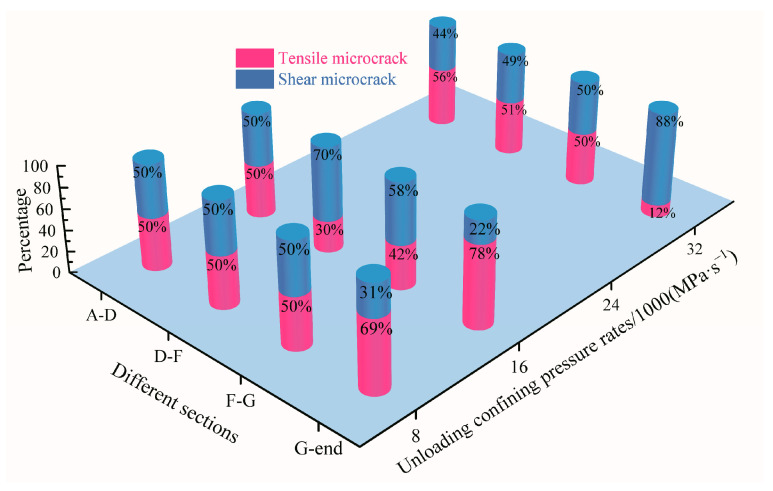
Percentage of tensile and shear microcracks in different damage sections of diorite under different unloading confining-pressure rates.

The distribution of *RA*–*AF* scattered points can closely reflect the evolution characteristics of microcracks in loaded rocks. Because the *RA* and *AF* thresholds of different rocks are variable, it is theoretically difficult to identify the boundary line of tensile–shear microcracks. For this reason, a two-dimensional Gaussian mixture model (TDGMM) was established by using MATLAB. According to the principle that a high *RA* value corresponds to shear cracking and a low *RA* value corresponds to tensile cracking, the TDGMM divides the microcrack evolution mode via two-dimensional cluster analysis. The percentages of tensile–shear fractures in different sections of diorite obtained based on the TDGMM under variable unloading confining-pressure rates are shown in Figure 13.

Figure 13 shows that the percentage of tension–shear microcracks in the A–D and D–F sections before the peak strength and in the F–G section after the peak strength of diorite under different unloading confining-pressure rates are all approximately 50%, indicating that the internal damage evolution mode of diorite under loading and unloading conditions is dominated by tension–shear mixing.

## 4. Nonlinear Damage-Mechanics Mechanism and Discussion

**Figure 14:**
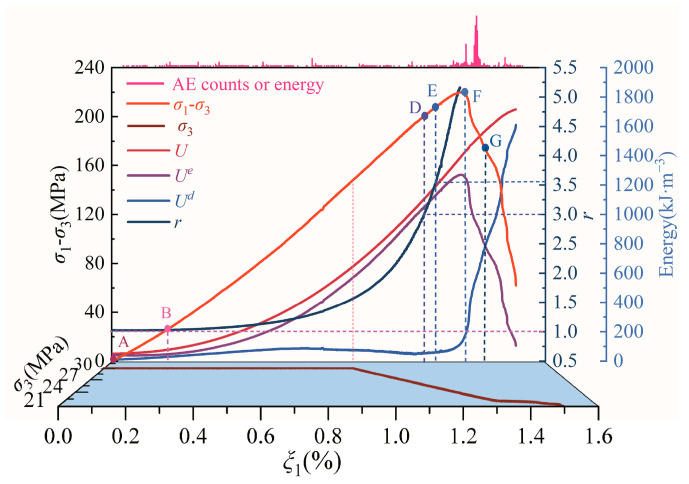
Damage evolution process of loading and unloading diorite.

Rocks are extremely complex materials. Macroscopically, they feature a large number of randomly distributed initial micropores or microcracks. Microscopically, there are heterogeneities in the spatial distribution of mineral particles. In terms of their mechanical behavior, rocks have different mechanical characteristics in different directions. As a result of these characteristics, the damage evolution process of rocks shows significant nonlinear characteristics. In the following, according to the nonlinear mechanical behavior of the strain energy sequence and the evolution characteristics of AE during loading and unloading, the internal damage evolution process of diorite may be divided into six stages (Figure 14).

1. Transcritical stability (A–B). During this stage, the strain-energy-promotion coefficient is *r* ≈ 1, and although the test apparatus inputs energy to the rock sample system, it is insufficient to break the equilibrium state of the initial rock system. The input energy further closes unclosed micropores or microcracks in the rock sample under hydrostatic pressure. If the confining pressure is gradually unloaded at any time during this stage, the strain energy stored in the rock sample will be released rapidly in the unloading direction, causing the closed micropores or microcracks to open gradually, such that the rock sample system will return to its initial hydrostatic pressure state.
2. Nonlinear stability (B–D). Due to the continuous input of energy from the testing machine to the rock sample, the strain-energy-promotion coefficient, *r*, becomes greater than 1.0 and shows a nonlinear increasing trend. At this time, the initial equilibrium state of the rock sample system has been broken. As a result of internal microdeformation, the rock sample continuously switches between equilibrium states; this change is accompanied by the storage and dissipation of strain energy. At this stage, the rock sample system enters a unique stable equilibrium state, indicating that the sample has good integrity, low internal damage, and small energy dissipation rates.
3. Period-doubling bifurcation (D–E). At this stage, the strain-energy-promotion coefficient, *r*, is greater than 3, the equilibrium state of the rock sample system is broken, and the attractor corresponds to multiple ordered equilibrium states. At larger *r* values, the number of ordered equilibrium states increases. During this process, the rock sample system undergoes a continuous period of doubling transitions between various equilibrium states under the action of internal microdeformation, accompanied by the storage and dissipation of strain energy. At this stage, although the unloading confining-pressure effect gradually releases a part of the stored strain energy, the total strain energy stored in the rock sample system continues to grow rapidly because the input energy to the rock sample system is greater than the dissipated energy. As a result of the larger strain energy, the self-repression of strain energy is active, and the active self-repression of strain energy causes increasing internal damage and energy dissipation of rock samples.
4. Weak chaos (E–F). When the strain-energy-promotion coefficient, *r*, in the rock sample system is greater than 3.57, the internal order state of the rock sample system is broken and enters a mixed state of order and disorder. During this process, the rock sample undergoes a complex mutual transformation between different states under the action of its internal microdeformation mechanisms, accompanied by a storage and dissipation of strain energy. This stage is also characterized by rapidly increasing internal damage and energy dissipation due to the greater self-repression of strain energy.
5. Strong chaos (F–G). With the gradual decrease of lateral constraints and the continuous input of external energy, the disorder of the rock sample system increases rapidly when the peak stress is passed, and the sample enters the post-peak-stress softening stage. During this process, a large amount of strain energy is consumed by the internal microdeformation mechanism of the rock sample system, such that the internal strain energy of the rock sample system is rapidly reduced, and its dissipated energy increases rapidly.
6. Failure (G–end). At this stage, the rock sample has failed, its bearing capacity decreases rapidly, the lateral restraint effect rapidly weakens, and the sample system has changed from a disordered state to a steady state.

The above division of the damage evolution process of diorite under loading and unloading conditions is based on the basic theory of nonlinear dynamics and chaos, which differ significantly from existing research results. Previous results suggested that the damage evolution process of a rock can be divided into five stages (crack closure, elastic deformation, crack initiation and stable crack growth, crack damage and unstable crack growth, and failure and post-peak behavior) according to the change in microcrack volume and characteristics during the process of loaded rock deformation [36,37,38]. Because rocks are extremely heterogeneous materials, their deformation, damage, and failure processes exhibit significant nonlinear characteristics, and their damage process and failure results are highly sensitive to the initial conditions and loading and unloading stress paths, the division of rock-damage evolution processes from the perspective of nonlinear dynamics can better reflect the essential characteristics of rock-damage evolution.

## 5. Conclusions

- The evolution of strain energy in diorite under loading and unloading conditions exhibits clear characteristics of order and disorder. At greater rates of unloading the confining pressure, the period-doubling bifurcation and chaotic mechanical behavior occur earlier in the damage’s evolution process.
- During the loading and unloading process, before the strain-energy-promotion coefficient is *r* < 3.57, the AE cumulative counts and AE cumulative energy changes are small. After the strain-energy-promotion coefficient is *r* ≥ 3.57, the cumulative AE counts and cumulative AE energy begin to increase rapidly, especially after crossing the peak stress value, and the growth rates become more rapid. On the other hand, when crossing the intersection of the strain energy and dissipation energy curves, their growth rates begin to decrease rapidly.
- During the diorite loading and unloading failure of diorite, the maximum Lyapunov exponent of its stress energy sequence shows a sudden phenomenon from positive to negative, and this phenomenon can be used as a distinguishing feature for complete failure of diorite.
- Before the loading and unloading peak strength, when the strain-energy-promotion coefficient, *r*, is equal to 1 or changes in the ranges of 1–3, 3–3.57, and ≥3.57, the rock’s strain-energy evolution shows the characteristics of supercritical stability, nonlinear stability, period doubling stability, and weak chaos, respectively. After loading and unloading peak strength, when the Lyapunov exponent of strain-energy evolution is >0 and <0, the evolution of the rock’s strain energy presents characteristics of the strong chaos and steady state, respectively.

## Figures and Tables

**Table 1:** Delay time interval and embedding dimension of the strain-energy sequence.

Unloading Confining-Pressure Rates (MPa·s^−1^)	0.008			0.016			0.032		
Different Sections of Stress–Strain Curve	E–F	F–G	G–	E–F	F–G	G–	E–F	F–G	G–end
Delay time interval	2	4	1	3	1	1	7	5	4
Embedding dimension	6	6	4	5	4	4	6	4	4

## Data Availability

The data presented in this study are available on request from the corresponding authors.
